# Does Patisiran Reduce Ocular Transthyretin Synthesis? A Pilot Study of Two Cases

**DOI:** 10.2174/1570159X21666230623094710

**Published:** 2023-09-25

**Authors:** Chiara Cambieri, Marco Marenco, Tania Colasanti, Carmine Mancone, Alessandro Corsi, Mara Riminucci, Laura Libonati, Federica Moret, Cristina Chimenti, Alessandro Lambiase, Fabrizio Conti, Matteo Garibaldi, Maurizio Inghilleri, Marco Ceccanti

**Affiliations:** 1 Department of Human Neuroscience, Centre for Rare Neuromuscular Disease, Sapienza University of Rome, Rome, Italy;; 2 Department of Sense Organs, Sapienza University of Rome, Rome, Italy;; 3 Department of Clinical Internal, Rheumatology Unit, Anesthetic and Cardiovascular Sciences, Sapienza University of Rome, Rome, Italy;; 4 Department of Molecular Medicine, Sapienza University of Rome, Rome, Italy;; 5 Department of Clinical, Internal, Anesthesiologist and Cardiovascular Sciences, Sapienza University of Rome, Rome, Italy;; 6 Cellular and Molecular Cardiology Lab, IRCCS L. Spallanzani, Rome, Italy;; 7 Department of Neuroscience, Mental Health, and Sensory Organs (NESMOS), Sant’Andrea Hospital, Sapienza University, Rome, Italy

**Keywords:** Amyloidosis, ATTR, ATTR-v, ocular, patisiran, RNA interference, siRNA, transthyretin

## Abstract

**Background:**

Variant transthyretin-mediated amyloidosis (ATTR-v) is a well-characterized disease affecting the neurologic and cardiovascular systems. Patisiran has been approved for neurologic involvement as it reduces hepatic synthesis of transthyretin (TTR). Eye involvement is a late-onset feature increasing the risk of glaucoma and cataracts in patients.

**Aims:**

The aim of this case series was to assess whether patisiran can effectively reduce TTR synthesis in such a barrier-protected organ as the eye.

**Methods:**

Two patisiran-treated ATTR-v patients underwent serum and aqueous humor sampling to measure TTR levels detected by SDS-PAGE and immunoblotting. Serum samples were compared to healthy control (HC), whereas aqueous humor samples were compared to non-amyloidotic subjects affected by cataracts and glaucoma.

**Results:**

Serum TTR levels representative of hepatic synthesis were sharply lower in treated patients if compared to the HC (-87.5% and -93.75%, respectively). Aqueous humor TTR levels showed mild-to-no reduction in treated patients compared to non-amyloidotic subjects with cataracts (-34.9% and +8.1%, respectively) and glaucoma (-41.1% and -2.1%).

**Conclusion:**

Patisiran does not seem to be as effective in inhibiting ocular TTR synthesis as it is in inhibiting hepatic synthesis. Re-engineering the envelope could allow the drug to target RPE cells thus avoiding any ocular involvement.

## 1 INTRODUCTION

Transthyretin-mediated amyloidosis (ATTR) is a systemic disease characterized by the deposition of amyloid fibrils in many different organs, such as the peripheral nervous system, heart, kidneys, vessels, joints, and eyes. There are two main different forms of ATTR, *i.e*. wild-type (ATTRwt) and hereditary (ATTR variant, ATTRv). In the former, which mainly affects the elderly, fibrils aggregate and are composed of a normal protein. In the hereditary form, fibrils are composed of a genetically-mutated protein with a high propensity to precipitate. More than one hundred pathogenic mutations are described for transthyretin (TTR), the most widespread being the Portuguese Val30Met variant (p.Val50Met). Inheritance of the disease is autosomal dominant, with incomplete penetrance [1].

Beyond other more common and studied manifestations, ocular involvement for ATTRv is described in many patient cohorts. 20% of ATTRv patients were diagnosed with glaucoma [2]; vitreous amyloid, neurotrophic keratitis, and tortuous retinal vessels were also described [3].

TTR is synthesized in the liver, the choroid plexus, as well as in retinal pigment epithelium (RPE) [4, 5]. The pigmented ciliary epithelium (PCE) was also demonstrated to synthesize TTR [6, 7], and 1.3 µg/ml of TTR was found in the aqueous humor [8]. In ATTRv, mutated TTR was found in the aqueous humor even after liver transplantation [9]. Indeed, the anterior and posterior eye chambers are immune-privileged target organs, protected by the blood-ocular barrier, which is functionally divided into a blood-retinal (BRB) and a blood-aqueous barrier (BAB) [10]. The inner component of both barriers is composed of tight junctions isolating the two chambers from immunoreactivity, circulating proteins, and drug delivery. TTR synthesis in the pigment epithelium and barriers can explain mutated TTR in the aqueous humor after liver transplantation in ATTRv patients [9]. TTR synthesis was demonstrated to decrease non-amyloidotic primary congenital glaucoma [11] and increase primary open-angle glaucoma [12].

Liver transplantation has demonstrated efficacy in prolonging survival and stabilizing neuropathy markers in ATTRv patients [13]. However, central nervous system and ocular complications may occur after the mean time patients would have survived without transplantation [14]. Conversely, livers from ATTRv patients are used in many transplantation centers to increase the number of liver grafts (domino liver transplantation), and patients receiving ATTRv livers may develop polyneuropathy [15-17] or amyloidotic cardiomyopathy [18, 19], though no ocular signs of ATTR were observed in different patient cohorts [20-22]. All these findings confirm that the effects of amyloidosis on the brain and the eye are not dependent on TTR synthesized by the liver but on TTR produced respectively by the choroid plexus and the eye.

Recent years have witnessed a surge of interest in ATTRv due to the recent approval of new effective treatments for the disease. Beyond the possibility to stabilize the TTR tetrameric structure [23, 24], new drugs using RNA interference [25] and antisense oligonucleotide [26] technologies are available to reduce TTR synthesis. In particular, patisiran uses lipid nanoparticles for nucleic acid delivery and to target hepatocytes following intravenous administration [27]. Patisiran exploits small interfering RNA (siRNA) technology to effectively reduce TTR translation [25]. Although the pharmacodynamics are widely known, naked siRNAs present several difficulties in terms of bioavailability due to blood nuclease activity and renal filtration resulting in rapid siRNA clearance. Moreover, its administration induces innate immune responses [28], and the polyvalent anionic and highly hydrophilic charge prevents cell uptake [29]. To solve the siRNAs drug delivery problem, patisiran is loaded into lipid nanoparticles (LNPs), in particular, heptatriaconta-6,9, 28,31-tetraen-19-yl 4-(dimethylamino)butanoate (DLin-MC3-DMA), 1,2-distearoyl-sn-glycero-3-phosphocholine (DSPC), cholesterol, and 3-N-[(ω-methoxypoly(ethylene glycol)2000)-carbamoyl]-1,2-dimyristyloxy-propylamine (DMG-mPEG-2000) at a molar ratio of 50:10:38.5:1.5 [29]. DLin-MC3-DMA is an ionizable lipid and is considered the gold standard for *in vivo* hepatic gene silencing [30]. The incorporation of DSPC into LNPs improves their stability during formulation and blood circulation [31]. DSPC, cholesterol, and DMG-mPEG2000 contribute to the stability of LNPs [32]. DMG-mPEG2000 is located on the surface of LNPs and is removed when apolipoprotein E (ApoE) coats the LNP and induces its endocytosis into hepatocytes mediated by the low-density lipoprotein receptor (LDL-R) [33].

Therefore, LNPs carrying patisiran target hepatocytes and need specific liver proteins to release their contents. The non-fenestrated endothelial cells interconnected through tight junctions of the blood-brain barrier (BBB), BAB, and BRB could be difficult for LNPs to overcome, even if the hydrophobic structure could allow LNPs to cross these barriers. Furthermore, ApoE and LDL-R, which are required for LNP endocytosis, seem to be expressed by RPE and PCE [34-38]. Although drug efficacy is reported in many studies [25, 39-41], the effects on barrier-protected organs are not described in depth. In particular, no clinical trial or case report has described the effects of patisiran on ocular involvement in ATTR. Tafamidis failed to halt the progression of oculo-meningeal amyloidosis in some ATTRv patients, probably due to its difficulty crossing BRB and BBB [42]. Other authors have reported an increase in the frequency of ocular abnormalities with increasing disease duration, regardless of therapy with tafamidis or liver transplantation [43]. However, the increased life expectancy of these patients due to new disease-modifying drugs makes it imperative to shed light on this aspect.

## 2 MATERIALS AND METHODS

### 2.1 Patient’s Medical History

#### 2.1.1 Patient 1

AC, 76 y.o., had a significant clinical history characterized by right carpal tunnel syndrome and previous surgery for aortic valve stenosis. He was admitted for acral symmetric paresthesia in the upper and lower limbs, confirmed as a sensory polyneuropathy by a nerve conduction study. A heart failure with preserved ejection fraction (HFpEF) with a concentric hypertrophic pattern and septal granular sparkling was found on the echocardiogram. Cardiac magnetic resonance demonstrated a late gadolinium enhancement, consistent with amyloid tissue. Genetic testing confirmed a Phe64Leu (p. Phe84Leu) mutation on the *TTR* gene also in two brothers. In 2019, the patient started patisiran at a standard dose. No ocular sign of amyloid deposits was observed at slit lamp examination. The patient developed a cortico-nuclear cataract in the right eye in 2020 with a progressive visual acuity decrease, leading him to cataract surgery the following year.

#### 2.1.2 Patient 2

EQ, 69 y.o., was admitted in 2007. His clinical history was significant for lower limb burning pain, associated with progressive distal atrophy in the upper and lower limbs, diarrhea, and orthostatic hypotension, with frequent lipothymic episodes and two events of syncope. He previously underwent surgery for bilateral carpal tunnel syndrome, with no regression of the symptomatology. Left ventricular hypertrophy with HFpEF was documented by an echocardiogram, together with a low-voltage electrocardiogram. A subcutaneous abdominal fat aspirate was stained with Congo red, demonstrating amyloid deposits. These findings and familiarity with sudden death led to genetic testing for ATTRv, which determined a Val30Met (p.Val50Met) mutation. The analysis was extended to family members, with his daughter being found to carry the same mutation. The patient underwent liver transplantation in 2011 and consequently started immunosuppressive therapy. In 2012, he underwent an experimental phase II study with doxycycline and tauroursodeoxycholic acid for 1 year. In 2015, he started treatment with tafamidis meglumine 20 mg/die. Due to the progression of the polyneuropathy, in 2019 he was switched to patisiran 0.3 mg/Kg/21 days. In September 2020, the patient underwent eye first aid treatment for acute pain and vision loss in the right eye. At ocular examination no sign of amyloid deposits was observed at the slit lamp examination, but intraocular pressure was 50 mmHg in the right eye and 24 mmHg in the left eye. Therefore, patient underwent glaucoma surgery Ex-PRESS^®^ (Alcon Laboratories, Fort Worth, TX, valve implantation).

### 2.2 Aqueous Humor and Serum Collection

A 0.1 ml sample of aqueous humor was collected from the right eye of both patients, using a 30G needle insulin syringe during planned surgery for ocular disease. The same amount of aqueous humor was also collected from a cataract- and a glaucoma-affected eye in non-amyloidotic patients as control samples. The amount of TTR in aqueous humor was demonstrated to increase in primary open-angle glaucoma [12], thus, a non-amyloidotic control sample was necessary. Serum samples were collected from patient 1 and patient 2 as well as from a non-amyloidotic healthy control (HC) to compare the effects of patisiran on hepatic and RPE /PCE synthesis.

The study was performed following the Declaration of Helsinki and its later amendments. Written informed consent was signed by each patient and by non-amyloidotic subjects.

### 2.3 SDS-PAGE and Immunoblotting

Protein contents of aqueous humor and plasma samples were determined by Bradford assay (Bio-Rad Laboratories, Hercules, CA, USA). Twenty μg of proteins were loaded and separated in NuPAGE 12% Bis-Tris Mini Protein Gels (ThermoFisher Scientific, MA, USA) by sodium dodecyl sulfate–polyacrylamide gel electrophoresis (SDS-PAGE) with MOPS running buffer, and electroblotted onto nitrocellulose membranes (Amersham Protan 0.45 µm, GE Healthcare, Little Chalfont, UK) by means of the Trans-Blot Turbo System (Bio-Rad Laboratories). In order to detect proteins on nitrocellulose membranes, the membranes were stained with Ponceau S reversible staining (Euroclone S.p.A., Pero, Milan, Italy), rinsed with phosphate-buffered saline containing 0.1% Tween 20 (PBS-T), and blocked in 5% non-fat dried milk (Euroclone S.p.A.) in PBS-T for 1 h at room temperature. After washings, the membranes were incubated with the anti-prealbumin (transthyretin) unmodified polyclonal antibody (Agilent Dako, CA, USA) at a dilution of 1:1000 in PBS-T containing 5% milk. Excessive primary antibody was removed by washing in PBS-T; the membranes were then incubated with horseradish peroxidase (HRP)-conjugate goat anti-rabbit IgG (Bio-Rad Laboratories) and diluted 1:3000 in PBS-T with 5% non-fat dried milk (Euroclone S.p.A.). Reactions were activated using the chemiluminescent HRP detection reagent Luminata Classico (Merck Millipore, Darmstadt, Germany). The total protein content obtained with the Ponceau S colorimetric method was used as a loading control for protein content. Quantification of protein expression was performed by densitometric analysis of detected bands (ChemiDoc ImageLab software version 5.1.2, Bio-Rad Laboratories). The protein expression level was quantified by densitometric analysis of detected bands (ChemiDoc ImageLab software version 5.1.2, Bio-Rad Laboratories) and reported as TTR/Ponceau S ratio.

## 3 RESULTS

Demographic data and results from patients and controls are shown in Table **1**.

### 3.1 Control Patients

The amount of TTR, expressed as TTR/Ponceau S ratio, was 0.032 in the HC serum, 0.095 in glaucoma aqueous humor and 0.086 in cataract aqueous humor controls.

### 3.2 ATTRv Patients

The amount of TTR, expressed as TTR/Ponceau S ratio, was 0.004 and 0.002 in patients 1 and 2 sera, 0.056 and 0.093 in patients 1 and 2 aqueous humor.

**Fig. (1):**
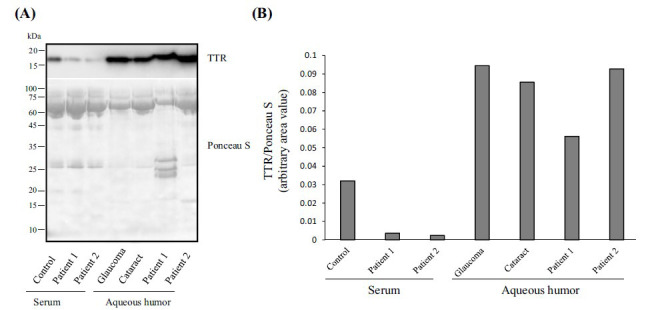
(**A**) Western blot analysis of serum and aqueous humor from patients and controls. (**B**) Densitometric analysis of serum and aqueous humor TTR levels from patients and controls.

Western blot analysis (Fig. **1A**, **1B**) demonstrated very low serum TTR levels in both treated patients if compared to the HC, only showing a small amount of residual protein in the serum of patients 1 and 2, which confirms previous data from the literature [25]. In particular, patients 1 and 2 displayed an 87.5% and 93.8% reduction, respectively, in serum TTR levels compared to the HC. Aqueous humor from the cataract and glaucoma controls showed similar TTR levels, with the latter displaying slightly higher values, in line with the literature (+10.5% compared to cataract control) [12]. Aqueous humor for patient 1 showed slightly lower TTR levels (-34.9% compared to cataract control), whereas aqueous humor in patient 2 was similar to cataract (+8.1%) and glaucoma controls (-2.1%).

## 4 DISCUSSION

The aim of the present study was to assess the possible effect of patisiran in inhibiting ocular TTR synthesis. While systemic effects of the drug, which was obtained by inhibition of hepatic TTR synthesis, have been fully described in many previous studies, there are many evidence gaps as concern the effects on barrier-protected organs. Some other therapies, *e.g*. liver transplantation and tafamidis, have failed to prevent ocular involvement [43]. Tafamidis demonstrated to cross the BBB and the blood-ocular barrier, with a ratio of TTR tetramer to tafamidis ≈ 2:1; however, only ≈ 1.5% of tafamidis in the plasma crosses the BBB on average and the drug was two-fold less concentrated in the vitreous body compared to the CSF [44]. The increased life expectancy of these patients suggests a higher prevalence of ocular and brain involvement in future years. The possible effect of patisiran on ocular synthesis was assessed in this small case series by evaluating TTR synthesis in the sera and aqueous humor of two patisiran-treated ATTR-v patients and comparing them to controls.

Serum TTR levels displayed an important reduction in treated patients compared to the HC, which is consistent with results illustrated in the APOLLO study [25]. In the aqueous humor, only one of the two patients showed a mild decrease in TTR levels, which was not comparable to the drastic reduction observed in the sera. These data, albeit evaluated in a very small sample, suggest a reduced/absent effect of patisiran in inhibiting TTR synthesis by the PCE as compared to the liver. This ineffectiveness could be attributed to the difficulty of the drug crossing the blood-ocular barrier and/or to the lack of endocytosis of LNPs containing the drug in the pigmented ciliary epithelium. However, LNPs are small hydrophobic particles and are expected to cross the blood-ocular barrier. Furthermore, patisiran failed to reduce TTR levels in the aqueous humor of the ATTR-v patient with glaucoma, which should increase barrier permeability [45]. Finally, ATTR-v patients displayed the loss of tight junctions and the fenestration of endothelial cells, at least at the blood-nerve barrier, with a loss of barrier functionality [46]. We hypothesize that the limited action of patisiran on PCE TTR synthesis could be attributed to reduced LNP endocytosis. Although ApoE and LDL-R expression is described in both PCE and RPE [4, 34-38], we suggest the presence of a reduced amount of these proteins in the PCE compared to the main organ involved in LDL clearance, *i.e*. the liver. In a recent paper [47], folate receptor was proposed as a possible target for retinal delivery of CRISPR technology, given the high expression of this ligand in retina [48]. This receptor could be considered for the PCE and RPE delivery of SiRNA targeting intraretinal TTR synthesis. Finally, no change in TTR levels was found in patient 2, who showed the most severe ATTR-v ocular complication despite therapy.

Some differences in terms of efficacy were also found between the two patients. The specific mutation (Val30Met *vs*. Phe64Leu) could play a significant role in barrier permeability, as well as the different age. In fact, blood-ocular barrier permeability was demonstrated to be age-dependant [49]; patient 1 was older compared to patient 2 and a better permeability is expected. Different ApoE and LDL-R isoforms between the two patients, allowing more or less LNP endocytosis into the PCE cells, could also be hypothesized.

These data need to be backed up by a larger cohort of patients, though the rarity of the disease and the difficulties obtaining biological material, such as aqueous humor, may represent a challenge to conducting large-sample clinical trials. Despite the small amount of available data, the drastic reduction in serum TTR levels in our patients confirmed a significant effect of patisiran on liver cells, even if the same efficacy was not confirmed by ocular epithelial cells, thus suggesting the need for drug remodeling to address this aspect.

## CONCLUSION

Patisiran seems to have limited-to-no effectiveness in reducing TTR synthesis inside the blood-ocular barrier. This finding, if confirmed by a larger cohort, could suggest the need to shape a new LNP envelope in order to allow drug endocytosis into PCE and/or RPE cells.

## Figures and Tables

**Table 1:** Demographic data and TTR/Ponceau S ratio in serum and aqueous humor of patients and controls.

**-**	**Sex**	**Age**	**Mutation**	**Serum TTR/Ponceau S**	**Aqueous Humor TTR/Ponceau S**
Patient 1	M	76	Phe64Leu	0.004	0.056
Patient 2	M	69	Val30Met	0.002	0.093
HC	M	72	-	0.032	-
Glaucoma patient	M	75	-	-	0.095
Cataract patient	M	69	-	-	0.086

## Data Availability

Not applicable.

## LIST OF ABBREVIATIONS

BAB: Blood-aqueous Barrier
BBB: Blood-brain Barrier
HC: Healthy Control
LNPs: Lipid Nanoparticles
PCE: Pigmented Ciliary Epithelium
RPE: Retinal Pigment Epithelium
siRNA: Small Interfering RNA
TTR: Transthyretin
